# 

**Published:** 1815-12

**Authors:** 


### MONTHLY CATALOGUE OF MEDICAL BOOKS.

An Essay on Dew, and several Appearances connected with it. By C. W. Wells, M.D. and F.R.S. of London and Edinburgh. The second Edition. 8vo. Taylor and Hessey.

An Epitome of the Second Edition of Hortus Kewensis, for the Use of Practical Gardeners; to which is added, a Selection of Esculent Vegetables and Fruits, cultivated in the RoyalGardens at Kew. By W. T. Acton, Gardener to his Majesty, with references to Figures of the Plants. 12mo. Longman and Co.

The American Medical and Philosophical Register; or Annals of Medicine, Natural History, Agriculture, and the Arts. Conducted by David Hosack, M.D. F.L.S. &c. &c. and John W. Francis, M.D. 4 vols. 8vo. Just imported. Callow.

A Letter on the Treatment of the Insane, with Remarks on the Nature, Causes, and Cure of Mental Derangement. By Thomas Bakewell. 8vo. Baldwin and Co.

The General Dispensatory, or Arrangement of the Pharmacopœias of London, Edinburgh, and Dublin. By S. Rootsey, F.L.S. 12mo. Baldwin and Co.

---

### *NOTICES TO CORRESPONDENTS.*

*We shall pay due attention to our anonymous Correspondent, and the work he has presented, when we sum up the various arguments concerning Contagion.*

Mr. Ronalds *will see that we have not been inattentive to his request. We always wish our correspondents to have every such facility.*

*We have been favoured with Communications also from* J. Atkinson, Edward Jackson, *Esquires;* A Surgeon-Apothecary, E. C. B., *&c. &c.*

---

ERRATA.

Page 363, line 41, for *affected* read *effected.*
—— 364, —— 22, for *unobserved* read *unobscured.*
—— 365, —— 39, for *preparing* read *proposing.*

### INDEX

### TO THE THIRTY-FOURTH VOLUME.

ABERNETHY's, Mr. analysis of his introductory lecture 65
——————, his promotion 349
Accoucheurs, on the necessity of 450
Acid, account of a new vegetable 165
——, danger of sulphuric 436
——s, their action in potassium 79
Adams, Dr. on the supposed obscurity of Mr. Hunter 29
———, on periodical blood-letting 110
Africa, pestilential winds of 409
Albers, Dr. his case of hydrophobia 426
Alimentary canal, inflammation of the 18, 100
Allen, Mr., on blood-letting in continued fever 249
Amaurosis, case of 425
Amiel, Mr. on the epidemic of Gibraltar 301
Amputation, on the English mode of 524
Andalusia, on the diseases of 406, 498
Aneurism, case of popliteal 116
———, case of axillary 118
———, on the treatment of 129
———, cases of 421
Animals, on the parts of 165
Aphonia, on 248
Apoplexy, on the causes of 112
Apothecaries' bill, passing of 169
——————, regulations respecting 251, 252
—————, abstract of the act concerning 344
—————, remarks on ditto 466
—————, hardship of apprentices to 346, 432, 466
Apprentices, letters from 432, 466
Armstrong, Dr., on nervous diseases 427
Arsenic, its use in cancer 198
———, on the cure of venereal ulcers by 348
———, effect of poison by 441
Arteries, influence of the nerves on he 54
———, on diseases of the 129
Arteries, on the pulsation of the 163
Assalini's, M., description of his surgical instruments 2
——————, successful use of 116
Athens, on the climate of 171
Atmosphere, on the state and composition of the 394, 467
Baillie, Dr., observations on his morbid anatomy 309
—————, president of the medical charity 523
Bakewell, Mr. on soda water 292
Balfour, Dr., on rheumatism 63, 196
Ballingall, Mr., on dislocated thumb 64
Battley, Mr. on vegetable extracts 293
Bedlam, evidence concerning 349
Black, Dr., memoirs of 36
Blood, on the vitality of the 29
Blood-letting, on periodical 110
———— in fever, on 249
———— unsuccessful in hydrophobia 426
Bone-nippers, new form of 243
BOOKS, Catalogue of Medical 88, 176, 264, 352, 440, 528
————, announcement of new 85, 172, 260, 352, 438
Botany, mode of teaching 258, 352, 438
Braconnot on animal fat 435
Brain, on the functions of the 23
———, case of a child born without a 104
———, case of diseased 519
Brown, Dr., on opium in ophthalmia 10
Brugmans, Professor, on hospital gangrene 394, 467
Brussels, state of the city of 349
Brydone, Mr., on the sirocco wind 408
Burnett, Gilbert, trial of 83
Buxton, Dr., on temperature in consumption 368
Cadiz, on the fever at 499
Calculous complaints, on magnesia in 445
Calculus concretions produce dumbness 172
———— found in a tumour 427
Calvert, Dr., on fever 158
Cancer in the breast, case of 150

### INDEX.

Cancer, use of arsenic in 198
———, remarks on 384
———, minutes of cases of 410
Capsule of the crystalline lens, on discovering the 453
Carlisle, Mr., on the parts of animals 165
Carmichael, Mr., on venereal diseases 226
———, on diseases supposed syphilitic 265, 454
———, on the review of his work 347
Cases—of chronic peritonitis 11
——— inflammation of the alimentary canal 18, 100
——— water in the brain 44
——— dislocated thumb 64
——— diseased liver 74
——— cataract 85
——— epilepsy 185, 517
——— malformation of the heart 101
——— diseased uterus 102
——— ovarian dropsy and hydrothorax 103
——— monstrosity 104
——— rupture 107
——— aneurism 116, 118
——— cancer in the breast 152, 154
——— hydrophobia 177, 426
——— of cold applications in gout 184
——— hydrocephalus 193
——— compression in rheumatism 196
——— venereal disease 233
——— pulmonary consumption 238
——— gonorrhœa 245
——— gleet 247
——— re-united finger 248
——— tænia ib.
——— polypus in the stomach 254
——— laryngitis 255
——— an enlarged tongue 257
——— fits 263
——— resembling syphilis and treated as such 265, 456
——— of a fœtus in the abdomen of a young man 317
——— febrile diseases treated with mercury 325
——— cancer 410
——— aneurism 421
——— amaurosis 425
——— tetanus 429
——— poison by arsenic 441
——— peritonitis 443
——— variola after vaccination 452
——— reproduction of a finger 515
——— ——— thumb 516
——— chronic hydrocephalus ib.
——— a premature child 517
——— sub-cutaneous tubercle 519
——— diseased brain ib.
Cataract, case of 85
Charms used in epilepsy ib.
Cheese, how to recover putrid 254
Chelwood, Somerset, singular occurrence at 225
Child, account of a piebald 172
———, born between the fourth and fifth month and reared 517
Chisholm, Dr., on the guinea-worm 57
Cold applications in gout, on 6, 184, 361, 522
Collectanea Medica 26, 114, 202, 301, 394, 467
Compression in rheumatism, beneficial effects of 196
Consumption, letters on 68
———, on temperature in 89, 368
———, on pulmonary 238
Contagion, observations on 337
Correspondents, notices to 88, 176, 264, 352, 440, 528
Critical Analysis of new works 43, 129, 226, 317, 410, 488
Cross, Mr., on the medical schools at Paris 478
Crowfoot, Mr., on poison by arsenic 441
Crutches, improvement in the construction of 128
Crystalline lens, on discovering the capsule of 453
Cupping, a substitute for leeches 64
———, on the art of 77
Cutaneous diseases, observations on 78
Davy, Sir H., on the action of acids on potash 79
Deafness cured by injections 525
Denman, Dr., on cancer 384
———, death of 526
Diarrhœa, case of obstinate 18
Dick, Mr., his account of a toad 169
Digitalis, its use in consumption 241
Diseases, on the conversion of 13
———, monthly reports of 87, 175, 263, 351, 528
——— considered as syphilitic 226, 265, 454
Dissections 11, 19, 73, 76, 104, 120, 145, 311, 320, 341, 437, 521
Distillation, mode of double 114
Donovan's, Mr., experiments on acids 164
Dropsy, case of ovarian 103
Drugs, London prices of 173, 261
Dublin, medical society instituted at 81
Dumbness, extraordinary instance of 172
Duncan's, Mr., case of tetanus 64
Edinburgh Medical and Surgical Journal, analysis of the 57, 243, 421
——— reviewers, answer to 29
——— medical society, state of 347
Emetics, their use in consumption 248
Entero-mesenteric fever, account of 338
Epidemic at Gibraltar, on the 301, 503
Epilepsy, charm for the 85

Epilepsy, on oil of turpentine in 248
——, case similar to 263
——, on the treatment of 517
Eruptions, remedy for tettery ones 257
Estlin, Mr., on amaurosis 425
Exeter, remarkable trial at 168
Extracts, on making vegetable 293
Farre, Dr., on the morbid anatomy of the liver 72
—— case of diseased arteries 131
Fat of animal bodies, on the 435
Fellowes, Sir James, on pestilential disorders 405, 498
Femoral artery, obliteration of 116
Ferriar, on the conversion of diseases 13
Fever, reflections on 158
——, on puerperal 183
——, at Guzerat 243
——, blood-letting in confirmed 249
——, on mercury in 32
——, on the entero-mesenteric 341
——, cases of 351
—— at Cadiz 499
—— at Gibraltar 503
Finger, re-union of the middle 248
Fits, remarkable case of 263
Fletcher, Mr., on gleet 247
Fœtus in the abdomen of a child 80
—— found in the abdomen of a young man 317
France, Royal Institute of 524
Galvanic battery, effects of a large 165
Gangrene, on hospital 394, 467
Gehlen, death and character of 438
George II. on the death of 140
Gibraltar, on the epidemic at 301, 503
Gibson, Mr., on the diseases of India 243
Glasgow, lectures in the university of 258
Gleet, case of obstinate 247
Gonorrhœa, remarkable instance of 245
Goodlad, Mr., on scrofula 65
Gout, on the cooling treatment of the 6, 184, 361, 521
Grimstone's, Mr., case of tetanus 427
Guinea-worm, account of the 57
Guzerat, on the fever at 243
Hæmorrhage, on the treatment of 21
Hæmorrhagia intestinalis, cure of 351
Hall, Dr., on a subcutaneous tubercle 519
Harmattans, account of African 409
Harveian oration, account of the 434
Head-ache, treatment of 351
——, case of 528
Heart, on rheumatism of the 85
——, malformation of the 101
Heat, double distillation from the same 114
Hepar sulphuris used in eruptions 257
Hermaphrodite, account of a 349, 525
Hey, Mr., on puerperal fever 13
Higgs, Mr., on the treatment of horses 420
Highmore, Mr., his case of a fœtus in a young man 317
Hodgson, Mr., on diseases of the arteries 129
Home, Sir E., on the brain 43—Remarks on ditto, 309—On the lampree and myxine 165
——, remarks on the opinions of 311
Hooping-cough, anecdotes relating to 127
——, prevalence of 351
Horse, case of one poisoned 437
Horses, on the management of draught 420
Hospital gangrene, causes of 394, 467
Hospitals, account of French 478
Hotel Dieu at Paris, account of 479
Hunter, Mr., on the supposed obscurity of 29—His opinion on the blood 30—On the doctrines of 65
Hydrocephalus, observations on 193
——, case of chronic 516
Hydrophobia, cases of 177, 426
Hydrothorax, case of 103
Index, announcement of the general 88, 440
India, on the diseases of 243
Infection, observations on morbid
Inflammation of the alimentary canal, case of 18, 100
Inoculation, observations on 295, 387
Instruments, description of new surgical 2
Intelligence, Medical and Philosophical 79, 163, 251, 344, 432, 522
Jackson, Dr., on inflammation of the alimentary canal 18, 100
——, on a case of malformation of the heart 101
Journal, artifice of a rival 1
Kelson, Mr., on cutaneous eruptions 78
Kinglake, Dr., on the cool treatment of the gout 6, 361
——, on morbid infection 97
——, on puerperal fever 183
——, on the obstetric practice 290
——, reply to 522
Klaproth, anecdote related by 437
Lampree, experiments on the 165
Laudanum, its use in ophthalmia 10
Laurence, Mr., on improved crutches 128
Lectures announced.—At the Theatre in Great Windmill-street, 172—Dr. Squire on Midwifery, ib.—At Glasgow, 258—At St. Bartholomew's Hospital, 259—At St. Thomas's and Guy's Hospitals, ib.—In St. George'sstreet, Hanover-square, and Gt. Windmill-street, ib.—At the Theatre of Anatomy, Bartlett's-court, Holborn, 260—At the Obstetrical Theatre, Berner's-street, ib.—Dr. Adams on the institutes and practice of medicine, ib.—Dr. Ramsbotham on midwifery, ib.—Mr. Charles Bell on surgery, ib. Dr. and Mr. Clarke on midwifery and female diseases, ib.—Dr. Squire on the same, ib.—Dr. Clutterbuck on the theory and practice of physic, ib.—At the Middlesex Hospital, 352—In Trinity College, Dublin, ib.—Mr. Carmichael on syphilis, &c. 438—Mr. Carpue on anatomy, ib.—Mr. Singer on electricity, ib.
Leeches, a substitute for 64
Lestes of Madeira, on the 408
Lettsom, Dr., death and character 526
Levanters, cause of 407
Linnæan Society, proceedings of the 81, 169
Lithotomy, instruments for 2
Liver, on the morbid anatomy of the 72
Londinensis, the reply of 17
Macartney, Dr., address to 82
Machell's, Mr., annular saw described 243
Mackesy, Mr., his cases of aneurism 421
M'Coy, Mr., on gonorrhœa 295
Madeira, on the Lestes of 408
Madmen, on the skulls of 316
Magnesia, its use in calculous complaints 445
Malformation of the heart, case of 101
Malic acid, experiments on 81, 165
Malis Dranunculus, account of the 57
Mapleson, Mr., on the art of cupping 77
Maton, Dr., his Harveian Oration 434
Medical and Philosophical Intelligence 79, 163, 251, 344, 432, 521
——— abuses in England 188
——— men, meeting of the society for the relief of their families 523
——— societies, accounts of 81, 348, 349, 523
Mediterranean, account of the winds in the 353
Mercury in tænia, use of 248
——— in febrile diseases 323
Metaphysics in medicine, on 161
Meteorological Register, 86, 174, 262, 350, 439, 527
Meyler, Dr., on calculous complaints 445
Monks, on the medicine of 122
Monster, account of a brainless 104
Moore's, Mr., history of the small-pox 121
Morbid infection, remarks on 97
Mucous membrane, on an inflamed 18, 100
Myxine, observations on 165
National vaccine establishment, report of the 166
———————, observations on the 367
Nerves, their influence on the arteries 54
Nervous diseases, on the treatment of 427
Nicaise, St., prayer to 124
Nicoll, Dr., on the fevers in India 244
Nippers, new form of bone 243
Notkerus, a monk and physician, account of 122
Obstetric practice, on 390
Ophthalmia, virtue of laudanum in 10
Opium in consumption, on the use of 241
Ovarian dropsy, case of 103
Paris, account of the medical schools of 478
Parry, Dr., on the pulse 80
————, on the arteries 163
Pemphigus, cases of 171
Peripneumony, case of 87
Peritonitis, on chronic 11
————, case of 443
Pharmacopœia Londinensis, account of 488
Phillips, Dr., on a remarkable fœtus 80
Physicians, anniversary of the College of 434
Piebald child, instance of one 172
Pinckard's, Dr., case of hydrophobia 177
Poison, action of vegetable 169
——— by arsenic, case of 441
———, effect of, on a horse 437
Polypus in the stomach, case of 254
Potash, action of acids on the hyper-oxymuriate of 79
Potassium, easy mode of procuring 26
Powell's, Dr., translation of the London Pharmacopœia, reviewed 488
Prichard, Dr., on epilepsy 517
Puerperal fever, on spirit of turpentine in 183
Pulse, on the nature of the 80, 163
Pyrosis, case of 351
Quackery, remarkable instances of 189
Reform, observations on medical 190
Report of diseases 87, 175, 263, 351, 528
——— of interesting trials 82, 168
——— of the National Vaccine Establishment 166
———————— apothecaries 251
———————— Small-pox Hospital 253
———————— Medical Society 523
Reproduction of a finger and thumb 515, 516
Rheumatism, on the pathology of 63
————, frequency of 87

Rheumatism of the heart, on the 85
———, remedy for 257
———, on compression in 196, 396
Rodman, Dr., on cancerous breast 150
———, his case of a child of premature birth 517
Royal Institute of France, proceedings of the 524
——— Society of London, meetings of the 79, 163, 524
Salisbury, Mr., his botanical establishment 258
Salter's, Mr., case of diseased brain 519
Sanchez's balsam, its efficacy in rheumatism 257
Satterley, Dr., death and character of 169
Saw, description of an annular 243
Scarlatina, frequency of 263
Scudamore, Mr., on aphonia 248
Scrofula, treatment of 65
Sea-water, its efficacy in gleets 247
Seringapatam, on the fevers at 241
Sewall, Dr., his case of diseased uterus 102
———, on arsenic in cancer 198
Sicily, profession of medicine in 186
———, on the climate of 353
Sirocco winds, observations on the 353, 404
Skulls, on the peculiarities of human 316
Small-pox, trials concerning the exposure of patients in 82, 83, 168
———, history of 121, 125
——— infection, extraordinary instance of 225
———, observations on 295, 387
——— Hospital, report of the 253
Smerdon, Mr., reply to 17
Soda water, on artificial 392
Southey, Dr., on consumption 238
———, chosen physician to the Middlesex Hospital 349
Speech, anomalous symptoms in the organs of 360
Spurzheim, Dr., in reply to Dr. Baillie and Sir E. Home 309
Starr's, Mr., case of peritonitis 443
Stewart, Dr., physician to the Westminster Dispensary 349
Stomach, polypus voided from the 254
Surgeon's Bill, account of 85
——— College, encomium on 192
Surgical instruments, new 2
Sutton, Dr., his letters on consumption 68
———, on the effects of temperature 87
———, reply to 368
Sympathy, on the doctrine of 66
Syphilitic complaints, resemblances to 265, 444
Tænia, case of 248
Temperature in consumption, on, 89, 368
Tennant, Mr., on potassium 26
———, on double distillation 114
———, memoirs of 202
Tetanus cured by tobacco 64
———, cases of 427
Tetters, remedy for 257
Thomson, Dr., his account of Dr. Black 36
Thumb, cases of dislocation of the 64
———, on the re-union of the 516
Toad found in a tree 169
Tobacco, its efficacy in tetanus 64
Tongue, case of enlarged 257
Trials, remarkable 82, 83, 168
Tubercle, case of subcutaneous 519
Tumour, calculus found in a 427
Turpentine, spirit of, its efficacy in puerperal fever 183
Ulcers, on the treatment of 21
Uterus, case of diseased 102
Vaccination, on the rejection of the bill for 113, 367
———, observations on 295, 387
———, occurrence of mitigated variola after 452
Vaccine Establishment, report of the National 167, 397
Variola, on mitigated 452
Vegetable extracts, mode of making 293
——— poisons, action of 169
Veins, on diseases of 129
Venereal diseases confounded with syphilis 226, 454
Vermes, on the organs of some 165
Vitality of the blood, on the 30
Vomiting, experiments on 524
Walker, Dr. J. K., on chronic peritonitis 11
———, Mr. R., on wounds and ulcers 21
———, on small-pox and vaccination 295
———, Dr. J., on the rejected Vaccine Bill 113, 367
Want, Mr., on Assalini's instruments 2
Ware, Mr., on opium in ophthalmia 10
Warren, Dr., on mercury in febrile diseases 323
Wayte, Mr., on accoucheurs 450
Welsh, Mr., on leeches 64
Whitbread, Mr. the elder, liberality of 410
Winds, on pestilential 353
Worcester, medical society at 349
Wounds, on the treatment of 21
Yeatman, Mr., on a spurious journal 1
———, state of medicine in Sicily 186

Yeatman, Mr., on the climate of Sicily 353

Yeats, Dr., on hydrocephalus 193

Yellow fever, on the treatment of 249

Young's, Mr., treatment of cancer, observations on 384, 41[illegible]

Young's, Mr., success of his plan at the Middlesex Hospital 526

Zinc, its use in consumptive cases 24[illegible]

### PLATES.

Mr. ASSALINI's new Surgical Instruments . . . . . 2
Mr. TENNANT's Tube for an easier Mode of procuring Potassium . . . . . . . . . . . 28
Mr. TENNANT's Vessels for Double Distillation from the same Heat . . : . . . . . . . . . 115
Mr. J. LAURENCE's improved Pair of Crutches . . . 128

